# Late systemic symptoms in head and neck cancer survivors

**DOI:** 10.1007/s00520-018-4577-3

**Published:** 2018-12-15

**Authors:** Elizabeth Wulff-Burchfield, Mary S. Dietrich, Sheila Ridner, Barbara A. Murphy

**Affiliations:** 1 Department of Medicine, Divisions of Medical Oncology and Palliative Medicine, University of Kansas Medical Center, 2330 Shawnee Mission Pkwy, MS 5003, Westwood, KS 66205 USA; 2 Center for Quantitative Sciences, Vanderbilt University School of Medicine, 2220 Pierce Ave, 571 Preston Research Building, Nashville, TN 37232 USA; 3 School of Nursing, Vanderbilt University Medical Center, 461 21st Ave South, Nashville, TN 37240 USA; 4 Department of Medicine, Division of Hematology/Oncology, Vanderbilt University Medical Center, 2220 Pierce Avenue, 777 Preston Research Building, Nashville, TN 37232 USA

**Keywords:** Head and neck cancer, Sickness behavior, Quality of life, Body image

## Abstract

**Purpose:**

Neuroinflammation and central sensitization from cancer and its therapy may result in chronic systemic symptoms (CSS) such as fatigue, sleep disturbance, chronic widespread pain, mood disorders, neuropsychiatric symptoms, and temperature dysregulation. We undertook a cross-sectional study of CSS in head and neck cancer (HNC) survivors to determine their frequency, severity, and impact.

**Methods:**

HNC patients without evidence of recurrence who were at least 12 months post-treatment completed a one-time battery of self-report measures including the Vanderbilt Head and Neck Symptom survey plus the General Symptom Subscale, the Body Image Quality of Life Inventory, Neurotoxicity Rating Scale, the Profile of Mood States, and a five-item quality of life measure.

**Results:**

One hundred five patients completed the surveys. Forty-eight point four percent of patients experienced one or more moderate-to-severe systemic symptom. The frequency of individual symptoms was between 20% and 56% with almost half of patients rating symptoms as moderate-to-severe in intensity. Low and high systemic symptom burden populations were identified. Previously undescribed chronic neuropsychiatric symptoms were also found to be frequent and severe. The vigor score on the POMS was low. Body image was not adversely impacted. At least 40% of HNC survivors have diminished quality of life, and up to 15% have a poor quality of life.

**Conclusions:**

CSS are common among HNC survivors and are frequently moderate to severe in intensity. Of note, previously underrecognized neuropsychiatric symptoms were endorsed by a significant cohort of patients warranting further study. Quality of life was diminished in a significant cohort.

## Introduction

The use of combined modality treatment, including surgery, chemotherapy, and radiation, has resulted in increased disease control in locally-advanced head and neck cancer (HNC) [1], but improved disease control occurs at the expense of increased acute and late effects from therapy. In the HNC population, acute tumor and treatment effects have garnered tremendous interest and have been extensively investigated. Until recently, late effects and their sequelae have been largely underrecognized and underappreciated. Improved treatment methodologies and the changing epidemiology, most notably the rise in HPV-associated oropharyngeal cancers, have resulted in a rapid increase in the number of HNC survivors. Accordingly, this expanding survivor population has generated a surge of interest in the late effects of HNC therapy. Evolving data demonstrate that acute toxicities may persist long-term and develop into late effects. In addition, late effects may manifest months or years after completion of therapy, persisting for years or even lifelong, far longer than previously believed [2, 3]. When severe, late effects may profoundly affect function and quality of life [4].

The most frequently studied late effects of therapy are those that are due to *local* tissue damage from cancer or its therapy. However, late *systemic* symptoms, which may have a more ubiquitous and profound impact on long-term function, have remained elusive from the standpoint of both research and management. Systemic symptoms, also known as sickness behaviors, include fatigue, central pain, neurocognitive dysfunction, mood disorders, thermal discomfort, sweating, gastrointestinal symptoms, and sleep disturbances. Systemic symptoms tend to occur in clusters, whichis felt to be due in part a common underlying pathobiology. While the mechanisms and pathways that contribute to systemic symptoms have yet to be fully elucidated, neuroinflammation is believed to be one of the important connective threads.

During the acute phase of illness or injury, the body must coordinate complex biologic pathways and behaviors in order to optimize the body’s response to disease and promote healing [5–7]. The illness response is mediated in part through peripheral pro-inflammatory and immune-activating cytokines which act as peripheral-to-central nervous system signaling molecules. The peripheral cytokines can induce a neuroinflammatory state and its associated systemic symptoms. Acutely, the illness response characterized by systemic symptoms such as fever, lethargy, and anorexia may be adaptive [8, 9]. However, if the inflammatory signal is overly exuberant or protracted, functional and anatomical central nervous system changes may develop. This may result in protracted or chronic systemic symptoms [9–11].

HNC and its treatment are both associated with elevations in peripheral pro-inflammatory cytokines [12, 13]. While the level of pro-inflammatory cytokines has been correlated with the grade of acute toxicity [14], the relationship between pro-inflammatory cytokines and late effects has not been reported. Furthermore, while available data indicate that head and neck cancer patients experience chronic systemic symptoms such as fatigue, anxiety, and depression [10, 15, 16], data describing the breadth, severity, and impact of late systemic effects are not available. To that end, we conducted a cross-sectional, observational, mixed-methods study in HNC survivors to determine the prevalence and impact of late systemic symptoms. Herein we report the results of the quantitative analysis.

## Materials and methods

### Patients

All patients were recruited from the Henry Joyce Cancer Clinic in the Vanderbilt-Ingram cancer center between November 6, 2014 and November 21, 2016. Patients included in this analysis were consented to participate in two clinical trials, the first of which included 92 patients and was entitled “Characterization of Chronic and Unremitting Symptoms in Long Term Survivors of Head and Neck Cancer.” The second study, entitled “Characterization of Chronic Systemic Symptoms among Participants in the Fibrosis-Lymphedema Continuum Study in Head and Neck Cancer,” included 13 patients who participated in an earlier, R01-funded study and had agreed to be contacted for participation in subsequent clinic trials. A convenience sample of 105 patients completed study measures and were included in the analysis.

Study eligibility criteria for both trials included the following: age 21 years or older, the ability to speak English, a history of histologically-proven HNC, completion of treatment a minimum of 12 months prior without evidence of recurrence. All eligible patients were approached and provided with information about the study. Interested patients were contacted by study staff and signed informed consent prior to completing study-related questionnaires.

### Methods

After signing informed consent, the participants completed the study questionnaires on an electronic web-based electronic data capture application (REDCap™).

### Questionnaires

#### Socio-demographic data form (self-report)

Captured birthdate, gender, race, ethnic category, highest educational level, marital status, employment status, area of residence, insurance coverage, and annual household income.

#### Disease and treatment data form (medical record review by study staff)

Captured data related to the patient’s cancer and treatment including diagnosis date, location, stage of disease, surgical treatment, medical oncology treatment, and radiation oncology treatment.

#### Patient-reported outcome measures

Patient-reported outcome (PRO) measures were included to address common local symptoms in the HNC population (Vanderbilt Head and Neck Symptom Survey version 2.0) as well as systemic symptoms (General Symptom Survey, Profile of Mood States-Short Form, Neurotoxicity Rating Scale). In addition, questionnaires were included to address body image and quality of life due to our interest in assessing the relationship between systemic symptoms and these outcomes.

#### Vanderbilt Head and Neck Symptom Survey version 2.0 plus General Symptom Survey (VHNSS v2.0 plus GSS)

The VHNSS v2.0 [17] assesses the prevalence and severity of treatment-related symptoms and their functional impact in patients with head and neck cancer. The VHNSS v2.0 consists of 50-items within 13 domains including nutrition, swallowing, xerostomia, mucositis, excess mucus, speech, hearing, taste change, smell, dental health, mucosal sensitivity, range of motion, and pain. Items are scored on a numeric scale rating the severity of the symptom from 0 (none) to 10 (severe). The VHNSS v2.0 takes approximately 10 min to complete. Cronbach’s alpha are > 0.9 in six symptom clusters and > 0.7 in the four remaining clusters [17, 18]. The GSS includes 11 additional items directed toward the systemic effects of cancer and therapy. Items are scored on a scale of 0 (none) to 10 (severe). The General Symptom Survey was specifically developed to assess the systemic symptoms associated with head and neck cancer and its therapy through review of the systemic symptom literature, patient interviews, and expert panel review. Content validity is being tested in an accompanying qualitative analysis to be published separately. Systemic symptoms investigated in this report include the items in the GSS plus two items from the VHNSS (“weight loss” and “loss of appetite”).

#### Profile of Mood States-Short Form

The Profile of Mood States-Short Form (POMS-SF) is a psychological evaluation tool used to assess mood states. This tool contains a 37-item scale consisting of adjectives rated on a 5-point Likert-like scale. It is composed of six subscales: depression (maximum possible score 28), vigor (maximum possible score 20), confusion (maximum possible score 20), esteem-related affect (24), tension (maximum possible score 24), anger (maximum possible score 24), and fatigue (maximum possible score 20). Cronbach’s alphas range from 0.78 to 0.91 [19].

#### Neurotoxicity Rating Scale

The Neurotoxicity Rating Scale (NRS) is a self-report measure examining neurocognitive symptoms associated with neurotoxicity of medical treatment. Its 37 items are symptoms rated in severity using a 5-point Likert-like scale bounded by “not present” and “extremely severe” [20]. Seven items from the NRS (restlessness, no interest in people, distractibility, irritability, decreased motivation, tension, and slowed movements) were chosen for inclusion in this analysis. Although the NRS has not been validated in the oncologic population, the selected items address unique symptoms that may be related to neuroinflammation and are absent in the other tools.

#### Body image quality of life inventory

The body image quality of life inventory (BIQLI) is a 19-item instrument which was developed to quantify the effects of body image on various experiences and life contexts [21]. Participants rate the impact of their own body image using a 7-point bipolar scale from − 3 to + 3, thereby permitting reports of negative, positive, or no impact [22]. Overall impact of body image can be determined by averaging the scores of all items. The Cronbach’s alpha of the scores in this study was 0.90 [21].

#### Quality of life

QOL was measured using two scales: a 5-item domain-specific QOL and a single-item self-anchoring scale [23, 24].

### Statistical analysis

SPSS version 24.0 was used for statistical analysis. Frequency distributions were used to summarize nominal data. Median and inter-quartile range (IQR) were used for continuous data summaries due to skewness of those distributions. Spearman’s rank correlation was used for determining the strength of the association between two global indicators of QOL. Two-step log-likelihood clustering using Schwarz’s Bayesian Criterion (BIC) was used to create groups of patients with similar levels of systemic symptoms.

## Results

### Patient and treatment characteristics

The sample was comprised of 105 patients. Available demographic and treatment characteristics are summarized in Table 1. Characteristics with some missing data are indicated in the table. Median age was 62.8 years. A majority of patients were male (74.3%) and Caucasian (94.2%). The most common primary tumor site was the oropharynx (57.1%) and a majority of patients had locally advanced disease, with 9.5% of patients diagnosed with stage III disease and 63.8% of patients with either stage IVa or IVb. A majority of patients received radiation-based therapy (*n* = 94 of 97 cases with information, 96.0%).

**Table 1:** Patient demographic and treatment data (*N* = 105)

Characteristic	Mean (SD) (min, max)
Age (*N* = 104)	61.9 (9.6) (37, 85)
Years of education (*N* = 104)	14.0 (2.3) (9, 20)
	*N* (%)
Gender	
Male	78 (74.3)
Female	27 (25.7)
Race (*N* = 103)	
White	97 (94.2)
Black/African-American	4 (3.9)
Other	2 (1.9)
Location of HNC	
Oral cavity	13 (12.4)
Nasopharynx	6 (5.7)
Oropharynx	60 (57.1)
Larynx	9 (8.6)
Other	7 (6.7)
Unknown	10 (9.5)
Stage at diagnosis	
Stages I and II	7 (6.7)
Stage III	10 (9.5)
Stage IV	67 (63.8)
Unknown	21 (20.0)
Surgery (*N* = 94)	
No	58 (61.7)
Yes	36 (38.3)
	*n*, median (IQR)
Time since completion of treatment (months)	98, 44.4 (18, 70)

### Prevalence and severity of systemic symptoms

The item pool assessed includes the 11 items from the GSS and 2 items from the VHNSS v2.0. The prevalence of systemic symptoms (score > 0) was high, with 6 out of the 13 items endorsed by more than half of participants: fatigue (52.4%), fatigue limiting day to day activity (50.5%), joint pain/muscle aches (53.3%), problems staying asleep (52.9%), sensation of cold (53.8%), and neurocognitive symptoms (53.4%). Another 4 out of 13 of the systemic symptoms were reported by between one third and one half of participants: anxiety (43.8%), problems falling asleep (41.0%), sensation of warmth (38.1%), and depression (38.1%). Weight loss was the rarest of the systemic symptoms, reported by 19.2% of participants. From the standpoint of intensity, 48.4% of patients experienced at least one systemic symptom of moderate-to-severe degree, as determined by a score ≥ 4.0 out of 10. Therefore, not only were symptoms frequent but also of clinically meaningful intensity.

### Clusters of systemic symptoms

**Fig. 1:**
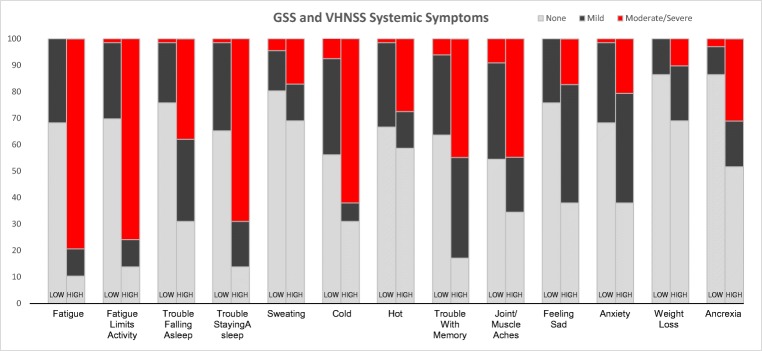
Vanderbilt Head and Neck Symptom Survey version 2.0 plus General Symptom Subscale by low and high systemic symptom patient clusters

A cluster analysis of patients with responses for all systemic symptoms (*n* = 95) was undertaken. This analysis resulted in the identification of two unique groups: a *low systemic symptom group* (*n* = 66 of 95, 69.5%) characterized by patients with none or very few moderate-to-severe systemic symptoms (none = 49 of 66, 74.2%, one = 12 of 66, 18.2%, two or three = 5 of 66, 7.6%) and a *high systemic symptom group* (*n* = 29 of 95, 30.5%) characterized by patients with at least two moderate-to-severe systemic symptoms (Fig. 1). Of note, 17 patients (58.6%) in the high systemic symptom group had 5 or more such symptoms. In the high systemic symptom group, the most common moderate-to-severe symptoms were fatigue (> 79.3%), difficulty staying asleep (69.0%), and cold (62.1%). Approximately 44.8% also reported moderate-to-severe issues with memory and joint pain or muscle aches.

### Body image

The median BIQLI score was 1.7 with an IQR of 0.7 to 2.5 indicating generally positive body image in the group.

### Neuropsychiatric dysfunction

The NRS responses confirmed the high prevalence of neuropsychiatric symptoms in this patient population (score > 0). The results are similar to those noted with the VHSS responses, providing convergent validity. Among the 7 neuropsychiatric symptoms included in this analysis, 6 were reported by between one-third to one-half of participants: restlessness (47.6%), tension (47.5%), decreased motivation (46.7%), distractibility (38.5%), slowed movements (38.5%), and irritability (38.5%). The remaining item, lack of interest in other people, was reported by 21.9% of participants. Among the positive responders, 45% indicated that at least one symptom was moderate-to-severe intensity (score ≥ 2). The highest prevalence reports at the moderate-to-severe level were for slowed movements (20.3%), lack of interest in people (11.3%), distractibility (16.3%), decreased motivation (18.8%), and tension (15.6%).

**Fig. 2:**
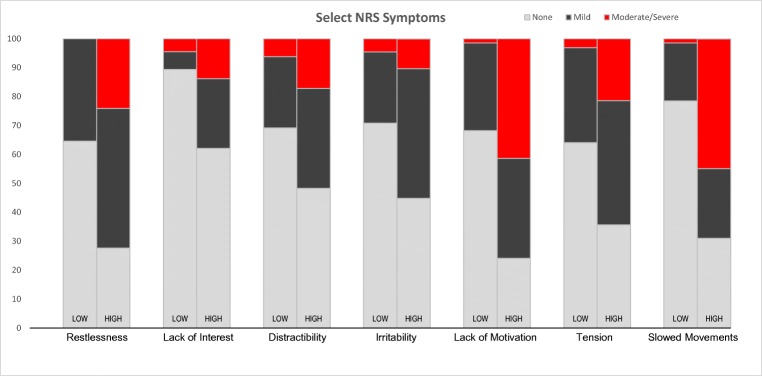
Unique items on Neurotoxicity Rating Scale by low and high systemic symptom patient clusters

Summaries of the NRS severity reports for the two systemic symptom cluster groups are illustrated in Fig. 2. There were striking differences in the prevalence of both mild and moderate-to-severe neuropsychiatric symptoms between the patient cluster with low systemic symptom burden and high systemic symptom burden. Of particular importance were the differences in the prevalence of both mild and moderate-to-severe “decreased motivation” and “slowed movements” between the two groups.

### Correlations among systemic symptom and neuropsychiatric symptom severity

**Fig. 3:**
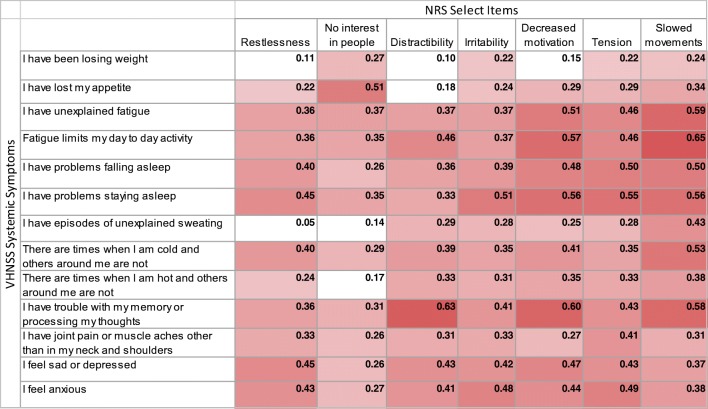
Correlogram between GSS items and selected NRS items

Correlations among the symptom groups are shown (Fig. 3). Gradations of shading indicate the strength of the correlations with the darker shades reflecting stronger correlations. Almost all correlations showed at least a minimally statistically significant correlation; those that fell below this threshold are depicted by a lack of shading (*r*_s_ < 0.20, *p* > 0.05).

### Mood

**Fig. 4:**
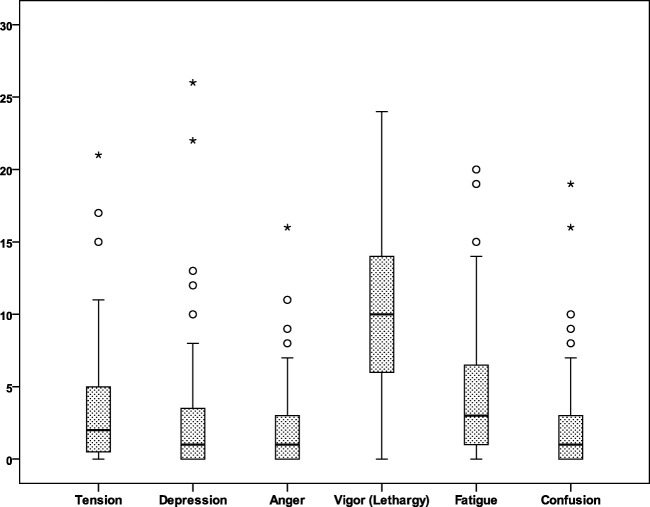
POMS-SF

Scores on the POMS-SF were generally within a more positive direction. For the negative emotions (fatigue, depression, confusion, tension, anger), most values were within the lower quarter of the possible range of scores. For vigor, the single positive emotion, the scores were in the mid-range of possible scores, see Fig. 4.

### Quality of life

**Fig. 5:**
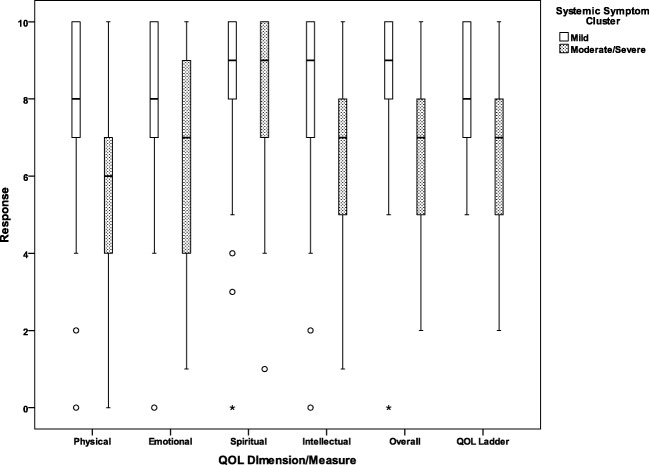
Single-item quality of life domains and global quality of life by low and high systemic symptom patient clusters

Finally, responses to the quality of life measures are summarized by systemic symptom cluster in Fig. 5. Patients in the high systemic symptom cluster demonstrated lower-median quality of life on all domains except for quality of life as compared to median quality of life for the low systemic symptom cluster. In addition to lower-median QOL, the high systemic symptom patient cluster was noted to have a higher proportion of patients rating quality of life as poor (scores of 0–4), respectively: physical 27.6% vs. 6.1% for the low systemic symptom patient cluster, emotional 27.6% vs. 6.1%, spiritual 6.9% vs. 6.1%, and intellectual 19.2% vs. 6.1%. Poor global quality of life was reported by 20.7% of those in the high systemic symptom cluster as opposed to 1.5% in the low systemic symptom patient cluster. A strong positive correlation was observed between the “global” domains on the QOL measure with the single QOL ladder (*r*_s_ = 0.65, *p* < 0.001).

## Discussion

As the number of HNC survivors increases, there has been growing interest in the quality of their survivorship. This includes a wide array of outcomes ranging from symptom burden and functional deficits to overall quality of life. Despite the rising interest in these topics, research in tumor- and treatment-associated late systemic symptoms has received little attention to date. To address this gap, we evaluated the prevalence of chronic systemic symptoms in HNC survivors as well as the association between chronic systemic symptoms and quality of life. Our findings demonstrated that a surprisingly high percent of long-term survivors experienced late systemic effects, with prevalence rates ranging from 25% to 40%. This is particularly striking when considering the population included in this study: HNC survivors at least one year out from completion of all cancer-directed therapy.

These late systemic symptoms are not only common but also severe. Our data demonstrate that approximately half of those reporting these symptoms rated the intensity in the moderate-to-severe range. It is, therefore, not surprising that chronic systemic symptoms were strongly associated with decreased quality of life, with patients in the high systemic symptom cluster rating median quality of life lower than in the low systemic symptom cluster in all domains except for spiritual quality of life. Interestingly, this finding mirrors the existing literature demonstrating associations between the burden of local symptoms and decreased quality of life [4].

In addition to lower overall median quality of life in the high systemic symptom patient cluster, our data demonstrate that 15% of HNC survivors reported poor global quality of life (rating quality of life ≤ 4/10). This particular cohort is likely unrecognized because much of the existing quality of life literature reports median quality of life [4]. This approach by its very nature fails to depict the range of participant responses. Furthermore, it fails to identify subsets of patients for who quality of life, symptom burden, or functional deficits are highly problematic. Given that traditional reporting methods for quality of life are frequently insufficient, more comprehensive statistical reporting methods should be used.

The cluster analysis is an innovative statistical approach that allows for the identification of subpopulations with distinct characteristics, including distinguishing symptomatology. By conducting a cluster analysis, we can evaluate and identify cohorts of patients with distinct features. In this study, we performed a cluster analysis that identified two discrete groups of patients: those with low and high levels of chronic systemic symptoms. Through this analysis, it was made clear that majority of patients who reported systemic symptoms experienced more than one. Although the taxonomy of chronic systemic symptoms continues to evolve, this phenomenon is frequently termed “central sensitization.” Multiple systemic syndromes are felt to manifest central sensitization, including fibromyalgia, chronic fatigue syndrome, irritable bowel syndrome, and others [25]. These conditions are marked by a high burden of systemic symptoms and “sickness behaviors” that cluster and give rise to the aforementioned well-defined syndromes. In recent years, there has been increasing interest in the biologic underpinnings of these conditions. Of note is that endocrinopathies are another proposed mechanism and should be ruled out by clinicians encountering patients with significant systemic symptom burden. However, recent data in the HNC population has demonstrated a relationship between fatigue and acute inflammation [26], lending further support to the theory of an inflammatory mechanism underlying many systemic symptoms. Furthermore, the non-oncologic literature has increasingly demonstrated that deranged interactions between the endocrine, immune, and neurologic systems result in systemic symptoms and sickness behaviors such as fatigue, depression, anorexia, widespread pain, and others [10, 11]. Therefore, it has been postulated that these symptoms may have a common underlying mechanism, and that the root of which may lie in neuroinflammation.

Neuroinflammation refers to a “cascade of altered neural activity that includes the induction of pro-inflammatory cytokines within the brain and spinal cord,” resulting in changes to cognition, pain, and even global function [9]. The biologic underpinnings of this state are still under investigation, but it has been proposed that this may indirectly result from elevated levels of peripheral (i.e., bloodstream) pro-inflammatory cytokines [9]. Even though peripheral cytokines cannot passively cross the blood-brain barrier, they can still communicate with the CNS through multiple mechanisms (e.g., active transport), whereby they can cause elevation of the levels of central pro-inflammatory cytokines. Elevations in central pro-inflammatory cytokines are associated with systemic symptoms, such as depression, anxiety, depression, cognitive changes, pain, and others [10, 11]. Owing to the inherent plasticity of the central nervous system, central neuroinflammation may result in irreversible changes to neural pathways [27].

Neuroinflammation and its effects are relevant not only to the phenomena of interest in this study (chronic systemic symptoms) [28] but also to the HNC patient population itself. There is mounting evidence that patients with HNC are at particular risk for neuroinflammation: HNC cell lines have been noted to release elevated levels of inflammatory mediators, and radiation to HNC in vivo has been also associated with increased levels of pro-inflammatory cytokines [12, 29]. These data suggest that both HNC tumors and their treatment can both contribute result in peripheral inflammation. Given the evidence that elevations in peripheral pro-inflammatory cytokines can lead to neuroinflammation and systemic symptoms, including in the HNC population [9], it would be reasonable to deduce that HNC and radiation-based treatment lead to neuroinflammation, but further research is needed to establish the relationship between HNC, radiation-based therapy, and neuroinflammation.

Systemic symptoms have been extensively reported in the general cancer population as well as specific cancer populations. Most reports address patients undergoing active treatment; however, increasing attention is being paid to the survivor population. Much of the literature describing chronic systemic symptoms in cancer survivors focuses on individual symptoms such as fatigue, depression, or neurocognitive changes, all of which have been well documented [30–32]. For example, neurocognitive effects of treatment have been extensively described in the breast cancer patient population [30, 33–35]. In addition to descriptive studies, multiple prospective, randomized studies have been undertaken employing physical [36, 37] and cognitive interventions [35], with varying results. Similarly, chronic fatigue has been documented in most cancer populations. For example, in a recent study of 275 rectal cancer survivors, 87% complained of “feeling worn out,” 85% complained of feeling “tired,” and 66% reported difficulty with sleeping [38]. To better understand the relationship between individual symptoms and underlying pathophysiology, a smaller but critical literature has emerged, exploring the clustering of systemic symptoms. For example, in a study of 74 newly diagnosed stage I to III breast cancer patients, using Bayesian network methods, investigators were able to identify that cognitive function was significantly influenced by sleep deprivation, thereby identifying a potential interventional target [39]. Select systemic symptoms, such as temperature dysregulation, remain poorly studied.

There are two unexpected findings from our study. The first of these was that patients expressed a positive body image. At first glance, this finding might seem counterintuitive, as one could expect that disfigurement associated with cancer and its therapy would create a negative body image. However, recent qualitative data indicate that HNC patients and survivors make efforts to disassociate themselves from the physical manifestations of their cancer, concentrating instead on functionality [40]. In this manner, a neutral or positive body image could be seen as part of a positive coping strategy. An alternative explanation for this finding could be that contemporary surgical and radiation techniques result in a lesser degree of damage to healthy tissues. For example, patients treated with radiation therapy with current techniques often note changes in the contour of their soft tissues without overt disfiguration.

An additional noteworthy finding was that the median score on the POMS-SF Vigor subscale revealed the HNC survivor population to be less energetic than the general population of cancer patients. The median vigor score of 10 in our study population indicates that the HNC survivors experience more lethargy and lack energy. Creating a point of reference for scores on the vigor score can be challenging. In one study in the oncologic population, the median vigor scores for depressed vs. non-depressed cancer patients were 9.98 and 14.37, respectively [41]. The impact of lethargy and motivation, a distinct but related construct, on function and quality of life warrants more extensive investigation.

While novel, this study is not without limitations. First, the cross-sectional nature of this study prevents study of the trajectories of chronic systemic symptoms. Therefore, further study is warranted to establish the trajectories of chronic systemic symptoms in the HNC population. This study also did not have a control population; however, the broad inclusion criteria effectively allowed for the approximately 60% of participants without chronic systemic symptoms to serve as a control group for those without. Despite the association between chronic systemic symptoms and quality of life, this study was not able to truly bring to light the impact of chronic systemic symptoms on HNC survivors. To that end, this study was paired with a qualitative analysis regarding impact, the data from which will be reported elsewhere the results of which are published separately. Finally, the NRS tool has yet to be validated in the oncologic population, thus further study is needed to validate it in this population; a cluster analysis could be beneficial to establish subscales.

Our study demonstrates the high prevalence and severity of chronic systemic symptoms in HNC survivors and the association between high systemic symptom burden and poor quality of life. Validation of these findings is needed. Optimally, prospective studies should be undertaken using patient-reported outcomes to capture systemic symptoms, objective measures of physical and cognitive function, and inflammatory biomarkers assessed at key time points along the treatment trajectory (baseline, during, and post-treatment). In addition, information regarding the pathobiologic underpinnings of systemic symptoms continues to emerge from the general medical literature. This work may inform ongoing and future work in the oncology population. A better understanding of mechanisms is necessary before interventional trials can be developed. In the meantime, HNC patients and their caregivers would benefit from pre- and post-treatment counseling regarding the chronic systemic symptoms.

## Abbreviations

HNC: Head and neck cancer
CSS: Chronic systemic symptoms
VHNSS-GSS: Vanderbilt Head and Neck Symptom Survey with General Symptom Subscale
POMS-SF: Profile of Mood States-Short Form
NRS: Neurotoxicity Rating Scale
QOL: Quality of life
